# Does digital collective learning improve with more participants? An experiment on a collective learning platform

**DOI:** 10.3389/fpsyg.2025.1605499

**Published:** 2025-08-05

**Authors:** Santos Orejudo, Oscar Casanova, Jacobo Cano-Escoriaza, Ana Cebollero-Salinas

**Affiliations:** Department of Educational Psychology and Sociology, University of Zaragoza, Zaragoza, Spain

**Keywords:** group size, collaborative learning, collective intelligence, speech, socioemotional competencies, social networks, interaction

## Abstract

**Introduction:**

The emergence of the Internet in the educational field has opened a significant number of possibilities, including interactive “virtual spaces” of collaboration in groups of many different sizes. Based on the principles of collective intelligence, our collaborative learning platform proposes an interaction model in which participants gradually reach solutions to a problem through a series of interaction processes that culminate in a step where consensus is reached.

**Methods:**

In our study, we compare results gathered from three groups of 11- to 12-year-old students (274, 56, and 69 participants) who dealt on the platform with a task related to emotional competencies in online environments. Large numbers of participants are possible on this platform thanks to its flexible design. Participants worked in seven phases to solve five questions with different answer formats based on a case study of social comparison on social networks.

**Results:**

Results reveal differences in terms of evolution according to group size: the largest group achieved the best results.

**Discussion:**

We analyzed the results through a series of variables that reveal further statistically significant differences among groups working on the same task in this novel learning environment.

## Introduction

The emergence and spread of the Internet have opened new spaces for learning with a series of conditions that differ somewhat from face-to-face environments. Referred to as the “digital space” (Pescetelli et al., 2021), this new context allows educators to create innovative, constantly evolving environments. Virtual connectedness, in turn, presents an ongoing challenge to education research. Participants can access immense quantities of information; moreover, they can respond to tests and obtain their results instantly. They can also become involved in new spaces for interaction under conditions considerably different from face-to-face interactions. Given this field’s relevance, we have launched the project “Computer Supported Collaborative Learning” with the aim of analyzing interactive contexts in digital (online) environments.

One of the most significant innovations introduced by this new “digital space” is undoubtedly the emergence of virtual collaborative learning environments. Such spaces allow us to transcend one of the main barriers associated with face-to-face interaction: namely, the number of people with whom we can learn together in teamwork. An online connection enables us to interact with many more people than we ever could in face-to-face interactions. Under such conditions, educators are faced with the novel task of guiding a large number of people in their learning experience. However, involving an elevated number of people in the same collaborative learning environment does not necessarily lead to an improvement in their learning. From a teacher’s perspective, the environment can condition the participants’ type of activity and methodology (Chimbi and Jita, 2021; Parks-Stamm et al., 2017; Sorensen, 2015) or it can lead to a work overload (Sorensen, 2015). From a student’s perspective, the fact of collaborating with a large number of peers can be associated with a series of negative consequences, such as lower individual participation levels (Cebollero-Salinas et al., 2022d; Gabarre-González et al., 2024; Siqin et al., 2015; Qiu et al., 2012; Shaw, 2013). These reflections lead us to reflect upon what we mean by the “number of participants in learning activities.” Analyzing the number of students taught by an educator in an online environment is not the same as analyzing the number of students involved in a collaborative learning-oriented task. Relevant conceptual studies have addressed the latter situation, particularly in contributions from neighboring disciplines, where the relevance of forming groups to resolve complex tasks was assessed and discussed (Mao et al., 2016). Group problem-solving tasks do have much in common with learning situations in general, and we shall see that constructs such as Collective Intelligence and Crow Intelligence can provide detailed analysis of processes we can then transfer to the field of learning.

An interesting proposal in this sense can be found in digital environments designed on the basis of the theoretical assumptions of Collective Intelligence. Within that theoretical framework, our research team (Orejudo et al., 2022; Bautista et al., 2022; Cebollero-Salinas et al., 2022c) has been addressing the double challenge of improving problem-solving processes by attempting to ascertain under which conditions collective work can outperform individual work while at the same time addressing eventual difficulties that tend to emerge when we increase the number of participants in a collective intelligence environment. One instance where the number of participants notably increases is when we transfer collective intelligence interactions to online environments. Under such circumstances, the construct of collective intelligence postulates that groups tend to outperform individuals in solving complex, vaguely defined problems, or problems that require a creative solution (Woolley et al., 2010). A series of experimental studies have indeed shown that small groups of 5–6 members can outperform individuals in addressing such problems (Woolley et al., 2010; Navajas et al., 2018; Toyokawa et al., 2019). One interesting hypothesis suggests that adding more members will improve solutions to such problems to an even greater extent. That hypothesis has given rise to new theoretical constructs, such as crowd intelligence and swarm intelligence (Yahosseini and Moussaïd, 2020). While certain advances have been achieved in the field of crowd/swarm intelligence, a series of accompanying difficulties have also become evident, most of which are associated with how groups function when they address tasks. Such difficulties include the “herd effect,” the polarization of responses, intense feedback loops, or an observed lack of activity on the part of individual participants (Toyokawa et al., 2019; Toyokawa and Gaissmaier, 2022; Yahosseini and Moussaïd, 2020). At any rate, these concepts are not equivalent, given that the collective intelligence construct assumes that participants interact to achieve solutions, whereas the condition of actual interaction among participants is not required in crowd or swarm intelligence.

One attempt to resolve the kind of interaction issues that tend to emerge in large groups involved in solving complex tasks can be seen in the Collective Learning Platform, https://ic.kampal.com/. Based on knowledge gathered in complex systems, this platform has been designed to overcome certain limitations that tend to emerge in large groups when they interact collectively (Orejudo et al., 2022). The platform can accommodate a large number of members (up to 5,000) who work on a task through successive work phases in groups of five “neighbors.” This imposed limitation ensures that each participant learns something from their “neighbors” while continuing to elaborate their own solutions to the issue at hand. In successive phases, a participant’s “neighbors” rotate (are interchanged) from phase to phase, thereby ensuring that each participant’s proposed solution is allowed the eventual chance to be copied and distributed throughout a network that emerges gradually through a series of nodes. This layout lets the system progressively determine each solution’s relative overall popularity. In a final consensus phase, the least frequent responses are deleted, and the most popular responses are shown to all participants. Table 1 describes the different working phases of the platform and its main features. Orejudo et al. (2022); Bautista et al. (2022); Cebollero-Salinas et al. (2024a,b) have described the platform’s mode of functioning in greater detail; those two studies also show how the Kampal platform can serve as a useful environment for solving mathematical problems and for dealing with personal/psychological/social issues. Other research teams have developed tools that, like ours, attempt to use the potential of online interaction in large groups to achieve learning experiences (Geifman and Raban, 2015) or as a means of problem-solving (Rosenberg et al., 2023, 2024). Along similar lines, Jeng and Huang (2019) designed a learning environment based on collective intelligence principles and oriented toward constructing mental maps. On a more conceptual level, Klieger (2016) addressed the potential of crowd wisdom for the educational sphere, transferring that theoretical framework to other disciplines with broader applications.

**TABLE 1:** Work phases on the platform.

Phase	Group	Edit items	Visual and copy scope	Dynamical items delete
1	Individual	Yes	No	No
2	Small group	Yes	Frozen 4 neighbors	No
3	Small group	Yes	Real time 4 neighbors	No
4	Small group	Yes	Real time 4 neighbors	No
5	Small group	Yes	Real time 4 neighbors	Yes
6	Big group	Yes	Top 10 Solutions	Yes
7	Big group	No	Top 10 Solutions	No

However, if we want to design such collective intelligence environments, we must consider several elements associated with the number of participants and the way interaction among them is organized. The starting assumption is that a small number of people who interact synchronously, either face to face or via a screen, are capable of achieving optimal conditions for task resolution, i.e., internal learning processes that lead to a solution for the problem at hand (Engel et al., 2014; Navajas et al., 2018; Hamada et al., 2020; Toyokawa et al., 2019; Woolley et al., 2010; Woolley et al., 2015). However, that very limitation does not allow researchers to explore what it is like to work with a larger group. When we prepare collaborative sessions in larger groups, we need to devote considerable thought to the type of interaction that will take place between participants; according to Yahosseini and Moussaïd (2020), such interactions can follow a variety of models ranging from synchronous to successive. Another possibility is to opt for an entire absence of interaction and apply an external method for aggregating individual responses. However, among all these models, the synchronous model is the only one that allows for the possibility of collectively achieving response consensus through a truly interactive environment where processes of interaction and social influence achieve an authentic learning effect. Large groups, however, are conditioned by a series of effects that limit their potential: mentioned above, these include the “herd effect,” the polarization of responses, an unacceptably wide variety of responses, intense feedback loops, or the fact that many participants simply do not take part sufficiently in the task (Toyokawa et al., 2019; Yahosseini and Moussaïd, 2020).

By analyzing interactive environments generated by collective intelligence models, we can achieve a theoretical framework that allows us to determine which factors possess the potential to optimize collaborative interaction. Woolley et al. (2015) differentiated among bottom-up and top-down factors. Bottom-up factors are the individual characteristics of the group’s members. A high degree of collective intelligence is associated with a high capacity for social perceptiveness on the part of individual members, i.e., their ability to recognize the emotions of others (Woolley et al., 2015). Another essential factor is a group’s degree of cognitive diversity. Homogeneous groups tend to exhibit less cognitive diversity (Aggarwal et al., 2019; Kozhevnikov et al., 2014); however, a greater degree of cognitive diversity promotes creativity and problem-solving, as it allows the group to apply a broader range of knowledge, skills, and perspectives. Still, it is not easy to demonstrate a direct relationship between diversity and performance, given that an increase in diversity can also give rise to conditions that can ultimately limit the process. Aggarwal et al. (2019) and Jeffredo et al. (2024) thus proposed an inverted U-shaped relationship between the variables of cognitive diversity and team learning performance. As opposed to bottom-up factors (reflecting individual characteristics of members), top-down factors are associated with group structure and the overall dynamics of interaction (Woolley et al., 2015). For collective intelligence to succeed, all members need to be justly and equally allowed access to communicate and participate; conversely, in groups where one or two individuals tend to predominate in group relationships, the other members will ultimately perform more poorly (Devine and Philips, 2001; Engel et al., 2014; Woolley et al., 2010; Woolley et al., 2015). Motivation and decision-making capacity are two additional factors that influence a group’s overall performance (Kerr and Tindale, 2004). Social influence and leadership phenomena within a team can affect its creativity (Becker et al., 2017; Toyokawa et al., 2019). Other studies have explored whether it is more beneficial for groups to consist of members who already know one another or whether *ad-hoc* -formed groups are better equipped to solve new tasks (Engel et al., 2014). Jeffredo et al. (2024) propose a three-level analysis of collective intelligence in small groups, similar to that of Woolley et al. (2015). This analysis considers macro factors influencing performance, micro factors related to group characteristics, and emergent factors related to group functioning. These factors include the type of task, communication, and social influence. These two approaches are useful for understanding group phenomena and their complexity.

Then, when the group task is designed with a learning goal in mind, we need to analyze not only the organization of interaction among participants but also the learning goals themselves. In this study, we designed a collaborative learning activity with the purpose of improving the socio-emotional competencies of participants in their use of social networks and digital devices (Cebollero-Salinas et al., 2022a,2022b). Planning such actions in a “virtual space” (see above) offers the advantage that the learning experience will take place in an online environment with specific characteristics that resemble social media and social networks, which, in turn, are the subject on which we asked participants to reflect on a socio-emotional level. Such online environments harbor a series of risks, including cyberbullying, Internet addiction, and the development of other habitual behaviors (Cebollero-Salinas et al., 2022a; Cebollero-Salinas et al., 2022b). Based on a case methodology, our specific task consisted in asking pre-adolescents to analyze a situation aimed at improving their degree of emotional independence on the Internet. They were asked to identify the advantages and disadvantages of the search for popularity and the gratifications that popularity can bring on social networks (Adjin-Tettey, 2022; Dhir et al., 2017; García-Jiménez et al., 2012), as well as the negative effect the search for popularity can have on problems faced by adolescents, such as social comparison, nomophobia (Topolewska-Siedzik and Cieciuch, 2018), and problematic smartphone use (Servidio et al., 2021). The task we proposed was particularly relevant for pre-adolescents, as one of the most noteworthy processes in the development of adolescent identity stems from the phenomenon of social comparison. Adolescence is a primary stage for the formation of identity (Topolewska-Siedzik and Cieciuch, 2018). Social validation processes play a major role in the way adolescents identify and define values, norms, and commitments (Erikson, 1971). The theory of social comparison propounded by Festinger (1954) provides one of the possible explanations for this phenomenon. Festinger’s theory describes peoples’ need to evaluate their opinions and capacities, as well as to validate their beliefs and attitudes, by comparing them with those of others. People compare themselves socially in several areas, including personality, wealth, lifestyle, and physical attractiveness (Jang et al., 2016). The comparison process fulfills several important functions: it regulates wellbeing and emotions, increases our self-esteem, helps us evaluate ourselves, and satisfies our drive for personal improvement (Liu et al., 2019). Certain authors differentiate between comparison of abilities and comparison of opinions. Comparison of abilities plays a key role in the construction of individual identity, as it provides information on personal accomplishments; this type of comparison can thus have a competitive character. On the other hand, comparison of opinions impinges upon attitudes, beliefs, and values regarding events and behaviors; it usually does not acquire the competitive, critical aspect inherent in comparison of abilities (Festinger, 1954; Suls et al., 2002).

Such identity negotiation processes are increasingly played out on social networks, where evaluation judgments regarding people’s competencies and abilities serve as important popularity indicators. Adolescents’ excessive tendency toward social comparison on social networks can entail risks and vulnerabilities for that age group. In university students, Vogel et al. (2015) found a higher level of social orientation and comparison, positively correlated with a greater use of social networks and negatively correlated with self-esteem and self-perception. In American university students, Lewin et al. (2022) found that the frequency of comparison of abilities was positively related to problematic use of the majority of social networks (Facebook, Instagram, Snapchat, TikTok, and Twitter); on the other hand, comparison of opinions was negatively related to problematic use of Facebook, Instagram, and Snapchat. In a study on adolescents, Geng et al. (2022) found a correlation between orientation toward social comparison and a more pronounced feeling of envy on social media by subjects who suffered from poor body image. Moreover, Fu et al. (2018) found that orientation toward social comparison was positively related to social competency and negatively related to depression when adolescents were competent and well-adapted. Further studies have found that comparison of opinions is positively associated with academic self-concept and positive social adaptation (Miao et al., 2018), as well as with self-esteem and materialism (Lins et al., 2016). In contrast, comparison of abilities is positively correlated with self-comparison, impulsive buying, and materialism (Lins et al., 2016), while it is negatively correlated with negative social adaptation (Miao et al., 2018). Pre-adolescents should thus learn how to develop and exert emotional competencies in online socialization processes.

Our study aimed to compare the evolution of three separate groups of students who were given the same task to solve. The task consisted of a scenario designed to improve social-emotional competencies for interacting in online environments: specifically, to identify social comparison mechanisms on the Internet in adolescence, and to acquire knowledge regarding the effects such comparison can have on emotional and personal wellbeing. As explained above, group size is a key factor in collective intelligence models (Takefuji, 2023): many questions remain to be answered regarding how group size can exert an influence on the result of an interactive process on an online platform such as the one featured in this study, which combines work in small groups with the dissemination of the ideas they generate across the entire network of users, culminating in the application of a consensus mechanism.

We hypothesized that we would observe different results depending on group size. Thus, we compared a large group (over 200 members) with two groups of medium size (approx. 60 members each). The experiment was conducted with three groups of students enrolled in primary education who carried out an activity on the Kampal Collective Learning platform.

## Materials and methods

### Participants

A total of 399 students in 5th and 6th grade (primary school) from the Autonomous Community of Aragon (Spain) participated in this study. They were enrolled in 12 centers that agreed to participate in our project devoted to the subjects of collective intelligence and harmonious online coexistence, promoted by Zaragoza University and the Department of Education of the Government of Aragon. Participation was entirely anonymous: data regarding sex and age were not collected. In Spain, the 5th and 6th grade academic years are attended by children ages 11–12.

All participating schools were invited to interact on the project platform under two different possible conditions: they could either join a series of sessions proposed by our research team or establish their own session schedule. Our objective, reflected in the first condition, was to gain the approval and participation of a maximum number of schools. We grouped participating subjects from those institutions into what we designated as the “Large Group” (Group 1), containing 274 individuals from 11 different schools. The remaining school established its own schedule, divided into two different groups: fifth-graders (*n* = 69. “Small Group 2”) and sixth-graders (*n* = 56, “Small Group 1”).

### Task

Collective Learning Platform, https://ic.kampal.com/ allows for a wide range of different tasks. For this study, we chose a learning task in which the educational tool was based on case methodology (Orejudo et al., 2008). Participants in all three groups were asked to analyze a hypothetical scenario. The protagonist was an adolescent girl who is a TikTok influencer with numerous followers. When one of her female friends launches a similar activity but obtains fewer followers, they enter into conflict, and the feud spreads to their families. The case, as presented, has two objectives. On the one hand, students are encouraged to develop a critical attitude toward the search for popularity on social media and social networks. On the other hand, they learn that comments on social media are only about external appearance and that they should therefore base their self-esteem on other elements apart from their body image. The case is designed to help them develop socio-emotional competencies that enable them to interact in online environments. The task was designed to be resolved in approximately 40 min. To carry out the activity, participants were asked to respond to 5 questions. Three were multiple-choice (Questions 1 and 2 had four options each; Question 3 had five options). The remaining questions, i.e., Questions 4 and 5, were open-ended (OE). The multiple-choice questions (Questions 1, 2, and 3) were graded according to the type of social-emotional skills they required: the most complex questions were given the highest scores. Thus, each answer had a value of 1–4 points. Students were allowed to write an additional text to justify why they had chosen a particular option. To assess the quality of these responses, we used the average syntactic breadth indicator (avg_max_depth), which refers to the depth of embedded “chains” of complement constructions sharing the same nominal head, including prepositional, adjectival and nominal modifiers (Brunato et al., 2020). This was calculated using the Profilind.ud application^1^.

### Statistical analysis

According to question type, we applied two separate statistical analysis strategies. For Questions 1, 2, and 3, we applied a repeated-measure design. Each participant received a score for each of the 7 activity phases on the platform. In each case, the response that was scored was the last one recorded by the respondent on the system at the end of each phase (as participants were allowed to record one or more responses or no response at all per phase). To avoid having too many lost values, participants who recorded no response at all during a phase were ascribed the response they had provided in the previous one. At any rate, only 3% of participants did not record any response at all during a phase. In those rare cases when they did not record a response, we chose to interpret that they were maintaining their response from the previous phase. This allowed us to apply a repeated-measure factorial design for each question for purposes of analysis. Independent variables were the group and the phase.

In this case (multiple-choice questions, i.e., Questions 1, 2, and 3), it was important for us to consider necessary conditions for the application of analysis of variance. Two especially relevant ones are multivariant normality and equality of variance/covariance matrices (Hair et al., 2007). If the sample size is large, the situation is less problematic if the first condition, multivariant normality, is not met (Hair et al., 2007, p. 364), which is what occurred in our case. To test the second condition, i.e., equality of variance/covariance matrices, researchers are advised to use Box’s M test. If equality is not shown, corrective measures need to be applied whenever the groups are of thoroughly different sizes, as would be the case in our situation. Thus, according to Hair et al. (2007, p. 363), if the more significant variances belong to the groups of larger size, the significance level should be raised. Conversely, when the greater variances belong to the smaller-sized groups, the significance level should be reduced to 0.03. In our case, the greater variances occurred in the larger-sized group: they could be observed in texts of a certain syntactical complexity. On the other hand, in the multiple-choice questions, the greater degree of variance occurred in Group 2; therefore, in the latter case, we increased the significance level to 0.08. Similarly, to compare measures in each repeated-measure group, we applied the Greenhouse-Geisser correction as an adequate alternative whenever sphericity could not be guaranteed. In this latter case, we used the SPSS program.

For the open-ended questions (OE), we followed a different analysis strategy.

Now we likewise chose *avg_max_depth* as a criterion for scoring written text answers. We applied this criterion of syntactical complexity to score Questions 4 and 5, as well as to evaluate the additional explanatory text we asked participants to provide for Questions 1, 2, and 3. In all these cases, the response rate for additional text was lower, as students were not only being asked to make a selection among multiple items, but to generate a text and record it in the system. (However, they were also allowed to copy a text written by one of their peers and present it as their answer in that phase.) Table 3 shows that their productions were considerably more diverse. Phase 1 produced an elevated number of text responses (461 responses, i.e., 1.15 responses per participant).

**TABLE 2:** Comparison by phases: multiple-choice questions (Q1–Q3).

	Fase														
		F1		F2		F3		F4		F5		F6		F7	
Group	N	Mean	± SD	Mean	± SD	Mean	± SD	Mean	± SD	Mean	± SD	Mean	± SD	Mean	± SD
**Question 1. Is it OK for Paula to try to become popular?**															
Large group	274	2.44	1.12	2.58	1.08	2.71	1.11	2.79	1.12	2.86	1.13	3.14	1.01	3.31	0.94
Small group 1	56	2.93	1.02	2.91	1.03	2.82	1.10	2.66	1.15	2.66	1.12	2.80	1.10	2.73	1.04
Small group 2	69	2.74	1.16	2.80	1.07	2.67	1.07	2.78	1.10	2.90	1.05	2.91	1.05	2.94	1.01
Total	399	2.56	1.13	2.67	1.08	2.72	1.10	2.77	1.12	2.84	1.11	3.05	1.04	3.16	0.99
**Question 2: Should Marta’s and Paula’s friends act differently?**															
Large group	274	2.83	0.83	2.90	0.80	2.93	0.80	2.93	0.81	3.05	0.70	3.16	0.56	3.16	0.54
Small group 1	56	2.41	1.11	2.50	1.08	2.43	1.16	2.55	1.14	2.38	1.18	2.36	1.17	2.43	1.14
Small group 2	69	2.54	1.02	2.61	1.00	2.55	1.02	2.52	1.08	2.51	1.09	2.49	1.15	2.49	1.15
Total	399	2.72	0.92	2.79	0.89	2.80	0.92	2.81	0.930	2.86	0.90	2.93	0.86	2.94	0.84
**Question 3. Should people only write positive comments under videos?**															
Large group	274	3.04	1.71	3.19	1.67	3.26	1.67	3.35	1.65	3.62	1.59	4.04	1.43	4.32	1.21
Small group 1	56	3.20	1.62	3.20	1.65	3.14	1.63	3.02	1.60	3.07	1.52	3.04	1.51	3.07	1.54
Small group 2	69	2.62	1.53	2.52	1.45	2.38	1.43	2.38	1.39	2.46	1.38	2.42	1.38	2.41	1.38
Total	399	2.99	1.67	3.07	1.64	3.09	1.66	3.14	1.64	3.34	1.61	3.62	1.57	3.81	1.50

**TABLE 3:** Comparison by phases: syntactical complexity of open response text under Questions 1–3.

	Large			Small 2			Small 1		
Phase	N	Mean	D.t	N	Mean	± SD	N	Mean	± SD
**Question 1: Is it OK for Paula to try to become popular?**									
1	204	3.70	0.13	9	3.13	0.54	15	2.72	0.40
2	63	4.07	0.19	12	3.60	0.48	15	3.59	0.41
3	47	4.38	0.22	13	2.99	0.46	15	3.03	0.41
4	43	4.92	0.23	7	4.12	0.57	16	3.06	0.39
5	177	4.33	0.14	8	4.20	0.57	11	3.12	0.49
6	126	4.24	0.16	10	3.81	0.49	22	2.53	0.35
7	45	5.31	0.22	5	3.55	0.68	9	2.97	0.51
**Question 2: Should Marta’s and Paula’s friends act differently?**									
1	189	3.09	0.11	7	1.93	0.50	13	2.52	0.33
2	47	3.15	0.16	9	1.81	0.44	14	2.85	0.31
3	40	3.57	0.17	10	3.60	0.39	12	2.37	0.34
4	44	3.91	0.16	5	3.41	0.51	12	2.42	0.34
5	150	3.38	0.11	5	5.04	0.53	6	3.07	0.46
6	118	3.56	0.12	10	4.36	0.40	21	2.43	0.29
7	47	3.71	0.16	3	4.95	0.63	8	3.27	0.39
**Question 3. Should people only write positive comments under videos?**									
1	187	3.88	0.12	10	2.72	0.46	14	2.36	0.41
2	46	4.07	0.18	11	2.51	0.46	9	2.36	0.45
3	31	3.91	0.21	8	2.94	0.49	6	3.01	0.57
4	35	4.37	0.20	1	3.43	1.03	6	2.60	0.59
5	123	4.29	0.14	5	2.91	0.70	7	2.87	0.52
6	130	4.37	0.14	15	3.00	0.42	11	2.38	0.46
7	44	4.69	0.18	2	2.82	0.81	2	2.79	0.98

However, the number of text responses was much lower in subsequent phases, thereby invalidating the possibility of applying repeated-measure ANOVA. We therefore chose, instead, to apply a linear mixed model for repeated measures (LMMRM) which allowed us to analyze variability among phases and among groups (fixed effects), while controlling the “participant” factor (random effect). By using that method, it was no longer necessary for us to have an answer from each participant in each phase. For this portion of analysis, we used the Jamovi program (Gallucci, 2019; The jamovi project, 2023).

## Results

Table 2 displays the results from the three multiple-choice questions, while Tables 3, 4 show the results of text response syntactical complexity from the two open questions, along with scores of the additional text students were asked to add to the first three questions to explain why they had selected a particular multiple-choice option. Major differences can be observed in terms of participant activity. For example, during the entire project, Questions 4 and 5 elicited a total of 1,219 and 1,269 responses, respectively (893 + 162 + 164, with a mean of 3.05; 903 + 178 + 188, with a mean of 3.18); on the other hand, the mean per participant and question on Questions 1, 2, and 3 was 2.18 (Q1, 705 + 64 + 103 = 872), 1.92 (Q2, 635 + 49 + 86 = 770), and 1.76 (Q3, 596 + 52 + 55 = 703; in this last case, the large group’s mean of activity was slightly higher). In other words, the students generated fewer responses to explain or justify their selection of multiple-choice item (Q1–Q3) than when they were asked to provide complete responses to open-ended questions requiring a text answer as the only option (Q4 and Q5).

**TABLE 4:** Comparison by phases: syntactical complexity of open response text under Questions 4 y 5.

	Large group			Small 2			Small 1		
Fase	N	Mean	D.t	N	Mean	± SD	N	Mean	± SD
**Question 4: Should we only half-believe what people say on social media?**									
1	362	3.40	0.11	35	1.67	0.34	64	1.90	0.25
2	101	3.95	0.20	27	2.13	0.38	17	2.47	0.49
3	63	4.73	0.25	25	2.67	0.40	17	1.96	0.48
4	61	4.56	0.25	14	3.54	0.54	20	2.67	0.45
5	169	4.14	0.15	33	2.16	0.35	27	2.86	0.39
6	104	5.04	0.19	24	2.18	0.41	16	2.65	0.50
7	33	5.81	0.34	4	2.88	0.99	4	3.34	1.00
**Question 5: Do you think that “likes” are the best goal to achieve on a social network?**									
1	324	3.90	0.12	35	2.13	0.33	70	1.90	0.25
2	119	4.61	0.17	33	2.72	0.34	22	2.59	0.41
3	65	4.76	0.23	30	3.32	0.36	15	2.17	0.48
4	53	5.33	0.25	13	3.12	0.51	12	3.49	0.53
5	196	4.61	0.14	34	3.27	0.34	36	2.28	0.33
6	98	5.10	0.19	27	3.50	0.38	28	2.79	0.36
7	48	4.90	0.27	6.00	3.87	0.73	5	4.90	0.81

Questions 4 and 5 (open-ended written text response).

We then compared the measures from Phase 1 to determine whether the point of departure was equivalent in all three groups. Multivariate results from Questions 1, 2, and 3 (Phillai’s trace, *F*_6, 790_ = 5.426, *p* < 0.001, η^2^ = 0.040) reveal differences among groups, confirmed for two of the three questions in the univariate statistics (Q1: *F*_2, 396_ = 5.514, *p* = 0.004, η^2^ = 0.027; Q2: *F*_2_,_396_ = 6.739, *p* = 0.001, η^2^ = 0.033; Q3: *F*_2_,_396_ = 2.225, *p* = 0.109, η^2^ = 0.011). In other words, a pairwise comparison of those two questions shows that Group 2 has a higher mean than Group 1; however, differences between Groups 1 and 3 and between Groups 2 and 3 are not significant.

In our analysis of answer syntactical complexity, we only found differences between groups in Phase 1 on Q3 (*F* = 4.48, *p* = 0.012, η^2^ = 0.041), P4 (*F* = 27.5, *p* < 0.001, η^2^ = 0.107), and P5 (*F* = 31.6, *p* < 0.001, η^2^ = 0.129). The descriptive results for these cases are displayed in Tables 3, 4: *post-hoc* comparisons reveal that, in all cases, Group 1 produced a more extended mean answer syntactical complexity than Groups 2 and 3. However, there were no differences between Questions 2 and 3.

**FIGURE 1:**
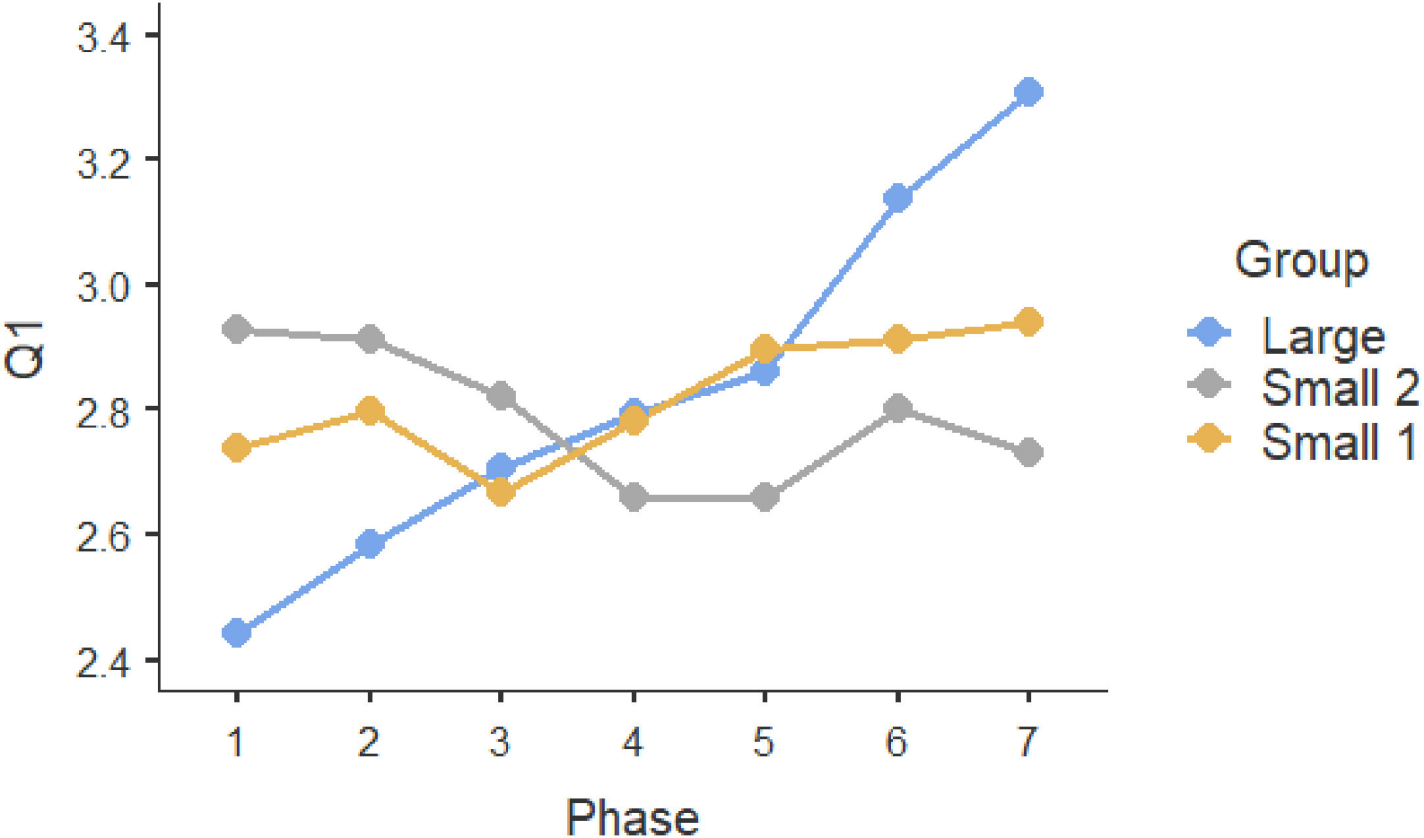
Question 1 multiple choice.

**FIGURE 2:**
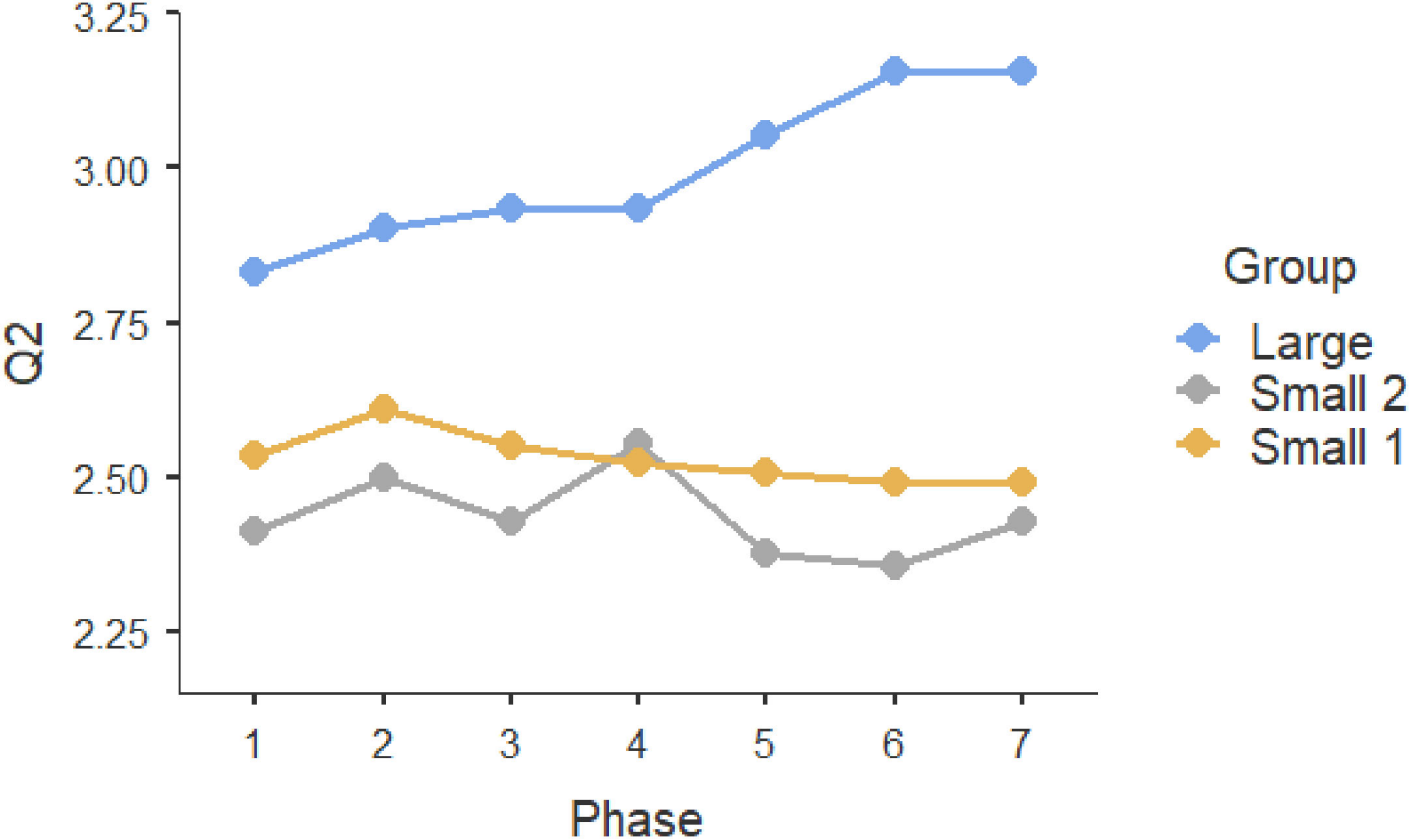
Question 2 multiple choice.

**FIGURE 3:**
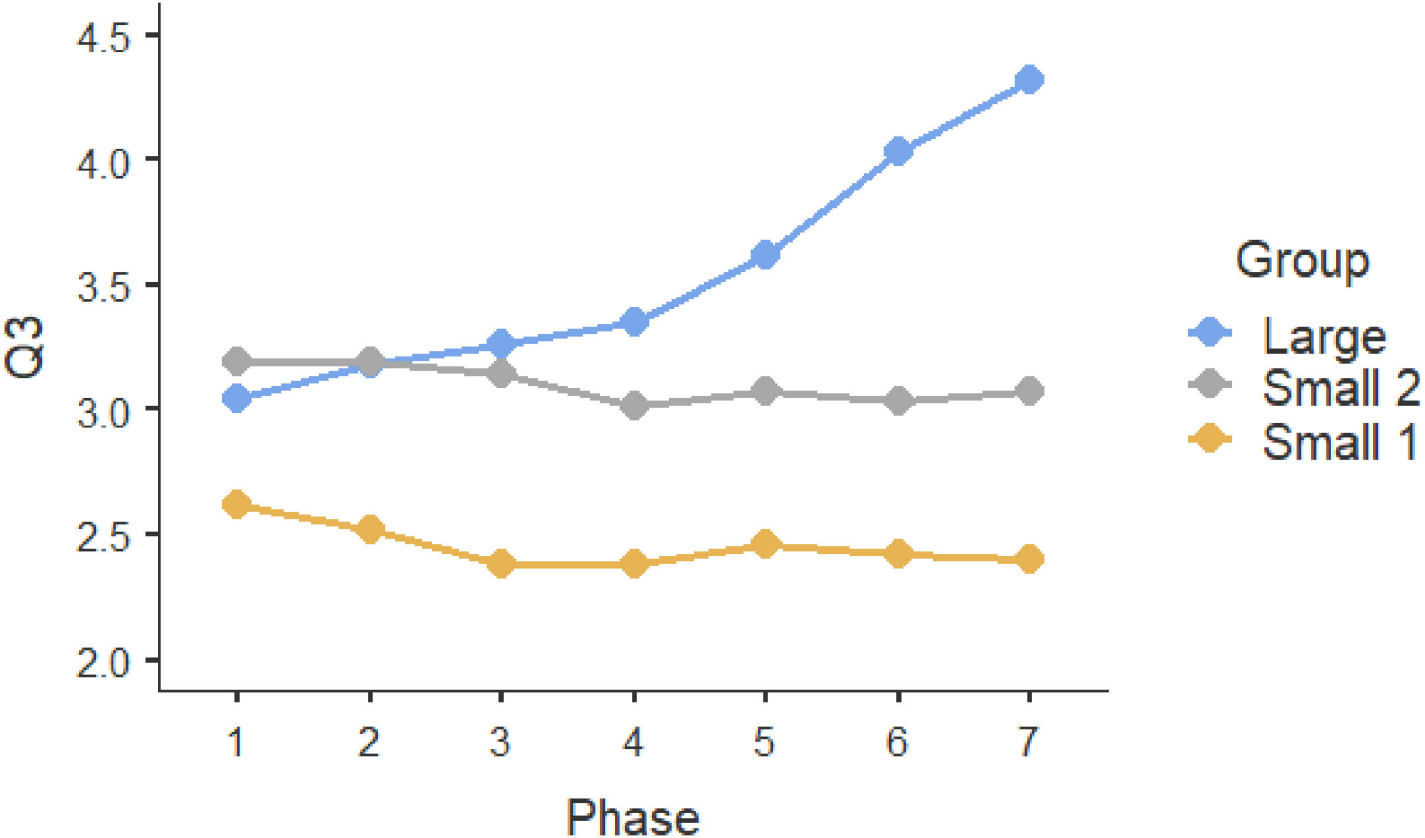
Question 3 multiple choice.

Repeated-measure ANOVAS show that participants evolved significantly along the seven phases of interaction in which they were involved. Statistically significant differences can be observed in the three initial questions, which were multiple-choice (Q1, *F* = 9.189, *P* < 0.001, η^2^ = 0.044; Q2: *F* = 4.881, *p* < 0.001, η^2^ = 0.024; Q3, *F* = 12.449, *p* < 0.001, η^2^ = 0.059). In all these three cases, the Large Group evolved along time (Q1, *F* = 58.966, *P* < 0.001, η^2^ = 0.178; Q2: *F* = 26.631, *p* < 0.001, η^2^ = 0.089; Q3, *F* = 57.219, *p* < 0.001, η^2^ = 0.173), but such change did not occur in the two smaller groups (Small Group 1: Q1, *F*_6, 171.858_ = 1.1492, *p* = 0.217, η^2^ = 0.026; Q2: F = 0.556, *p* = 0.630, η^2^ = 0.010; Q3, *F* = 0.414, *p* = 0.737, η^2^ = 0.007; Small Group 2: Q1, *F*_6, 259.846_ = 1.329, *P* = 0.261, η^2^ = 0.0019; Q2: *F* = 0.319, *p* = 0.758, η^2^ = 0.005; Q3, *F* = 0.913, *p* = 0.414, η^2^ = 0.003). These results can be observed in graphs in Figures 1–3. *Post-hoc* differences reveal statistically significant progressive changes in the Large Group. In Question 1, changes occurred in all pairs we compared, except in the transitions between Phases 3–4 and 4–5; in Question 2, the four initial phases were not significantly different, but changes emerged in Phases 4–6, then no further changes occurred between Phases 6 and 7. In Question 3, there were no differences in the three initial phases, but statistically significant changes occurred in all subsequent phases.

**FIGURE 4:**
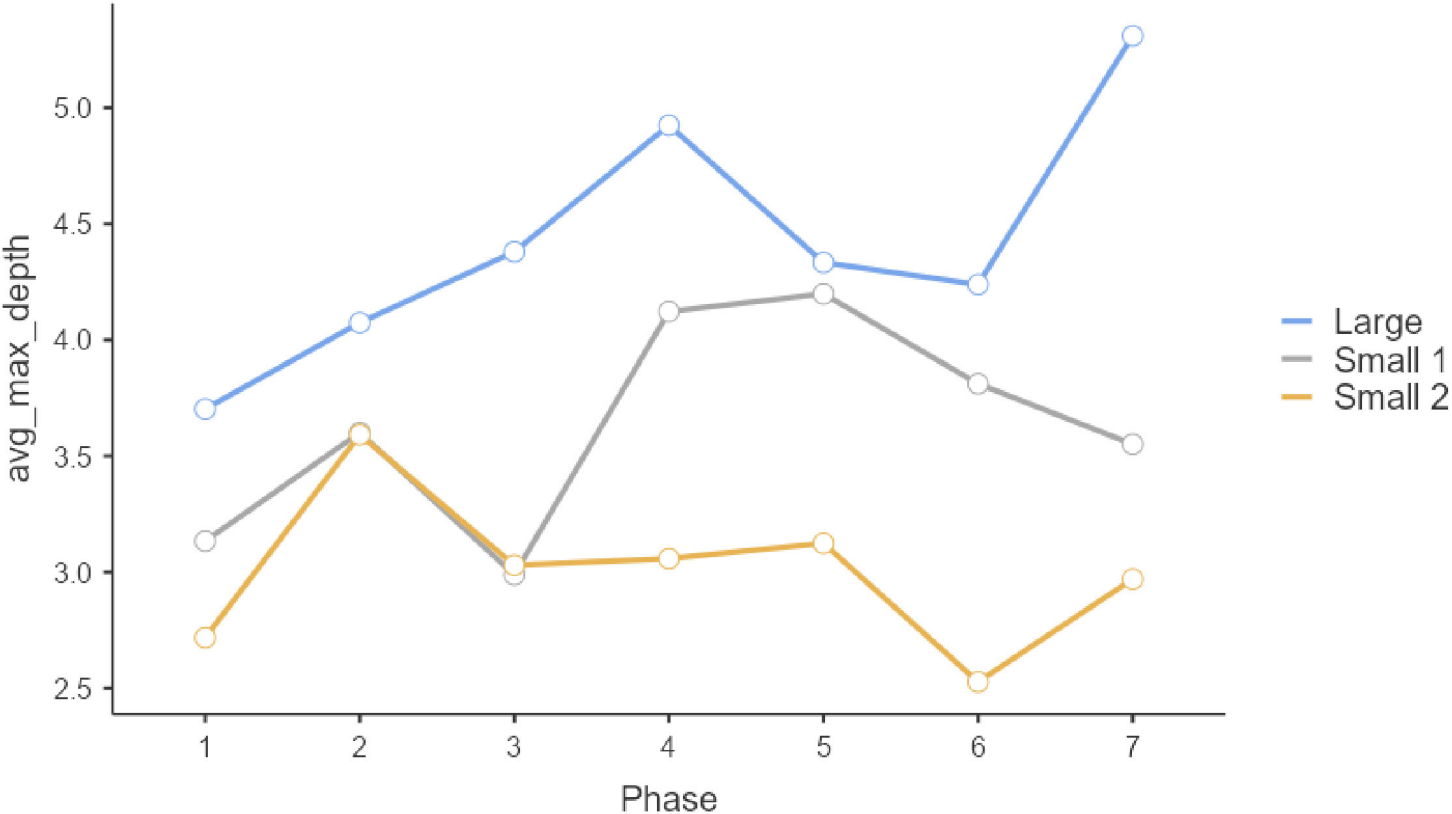
Question 1 syntactical complexity.

**FIGURE 5:**
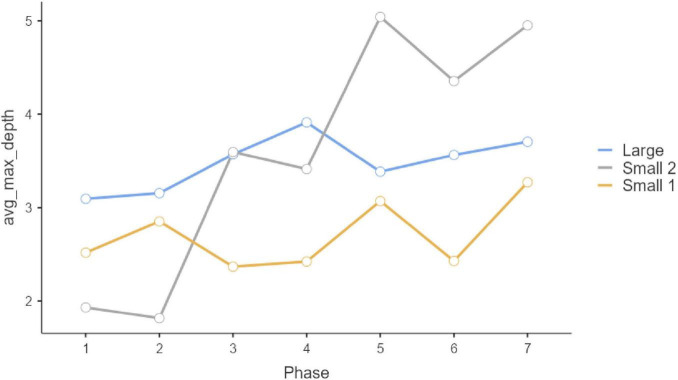
Question 2 syntactical complexity.

**FIGURE 6:**
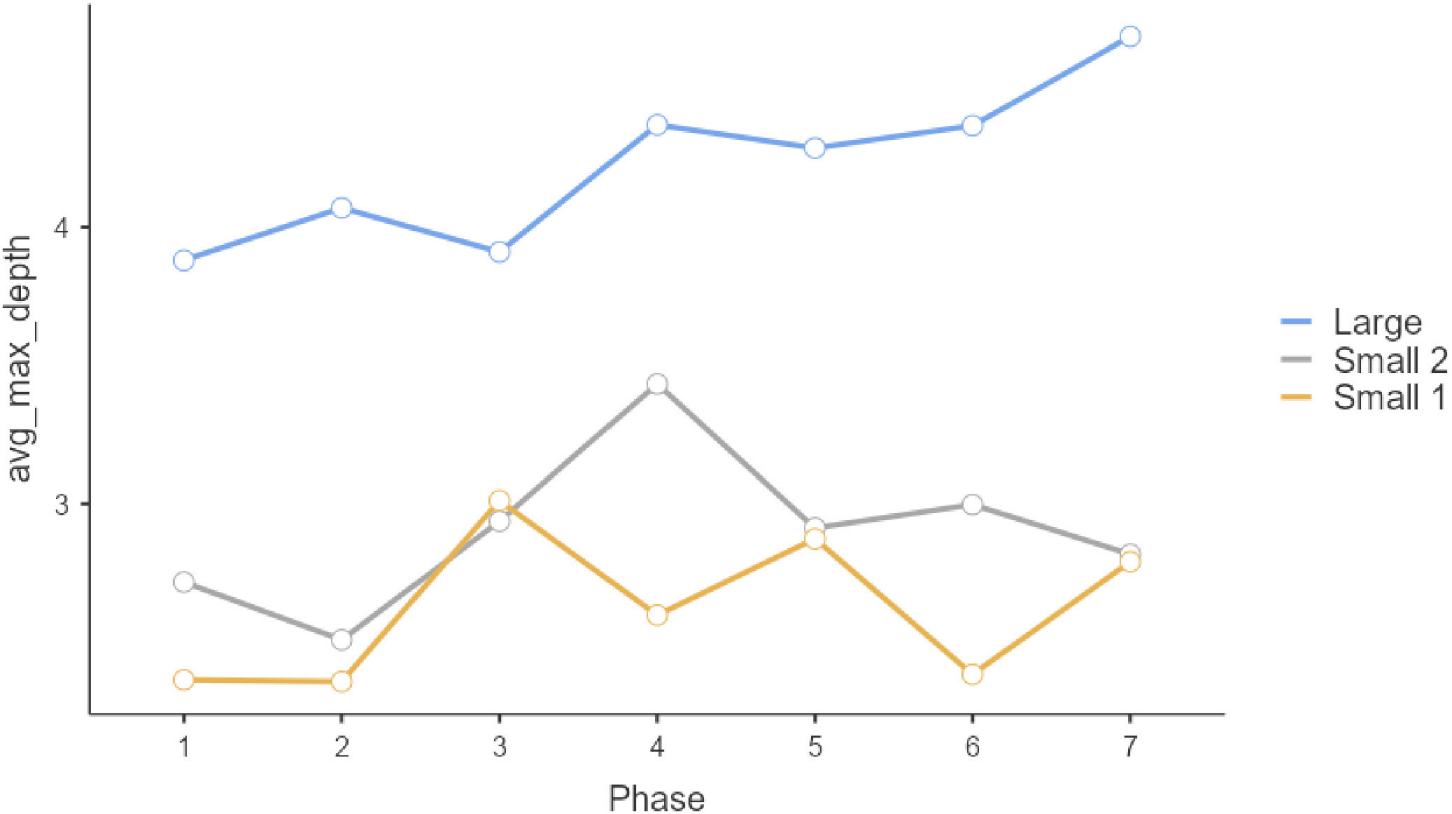
Question 3 syntactical complexity.

Regarding the complexity of the additional text answer written to justify the selection of multiple-choice option in Questions 1, 2, and 3 (Table 3; Figures 4–6), we observe differences among groups in all three questions (Q1: *F* = 14.51, *p* < 0.001; Q2: *F* = 6.34, *p* = 0.002; Q3: *F* = 16.186, *p* < 0.001). Similar to trends we had observed regarding the number of responses per participant, here we also ascertained that Group 1, in all three cases, provided complex responses (on average) than Groups 2 and 3 on Questions 1 and 3. Meanwhile, on Question 2, Small 1 obtained a lower score than Large and Small 2 (*Post Hoc* Tests Holm comparisons).

Phase-group interaction on Question 1 and on Question 3 is not statistically significant (Q1: *F* = 1.55, *p* = 0.101; Q3: *F* = 0.410, *p* = 0.996), however, it is significant in Question 2 (Q2: *F* = 3.72, *p* < 0.001). This interaction effect can be observed in Figure 2A. Thus, in terms of progress along phases, Large group had a slight tendency to increase its score between Phase 2 and 3 and 4 and 5, whereas Small 2 show significant differences among phases 2 and 3. Small 1 no show significant differences among phases. There is no phase effect on Question 1 and 3 (*F* = 2.07, *p* = 0.054; *F* = 0.70, *p* = 0.649).

Taken together, the three models estimate a substantial percentage of explained variance (Q1 R^2^-_*Marginal*_ = 0.095; Q2 R^2^-_*Marginal*_ = 0.050; Q 3 R^2^-_*Marginal*_ = 0.104). In all three cases, incorporating the random effect of participants turns out to be statistically significant (Q1: *likelihood ratio test* AIC = 3,518, *p* < 0.001; Q2 AIC = 305, *p* < 0.001; Q3: AIC = 2802, *p* < 0.001). The scores of the intraclass correlation coefficients are clearly related among themselves (Q1: ICC = 0.579; Q2: ICC = 0.487; Q3: ICC = 0.754).

**FIGURE 7:**
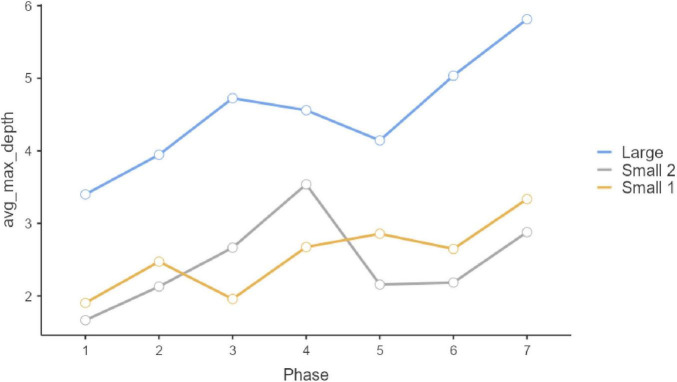
Question 4 syntactical complexity.

**FIGURE 8:**
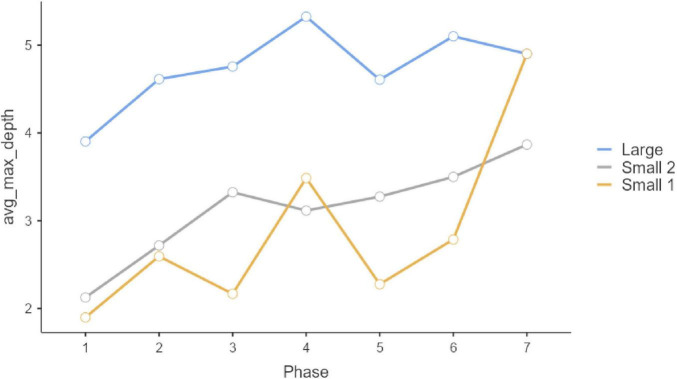
Question 5 syntactical complexity.

Questions 4 and 5 are substantially and qualitatively different from Questions 1, 2, and 3, as they exclusively consist of freely composed response text. Here, as explained above, we analyzed syntactical complexity. As in the previous case, a significant main effect is exerted by the Group factor (Q4: *F* = 62.04, *p* < 0.001; Q5: *F* = 40.86, *p* < 0.001). Here, once more, the large group (Group 1) achieved the highest score (Figure 7). Regarding the criterion of evolution along phases, phase-group interaction was not statistically significant (Q4: *F* = 1.27, *p* = 0.228; Q5: *F* = 1.09, *p* = 0.363), but evolution along phases was significant (Q4: *F* = 5.65, *p* < 0.001; Q5: *F* = 9.22, *p* < 0.001, see Figure 8). In that case, the phase factor played a determining role in transition differences between Phase 1 and Phase 2. In other words, the responses in phase 1, individual, are less complex than those in phase 2 and subsequent phases. Taken together, the models we estimated for these questions explain a substantial percentage of variance (Q4 R^2^-_*Marginal*_ = 0.197; Q5 R^2^-_*Marginal*_ = 0.187). In all three cases, the incorporation of the random effect “Participants” is statistically significant (Q4: *likelihood ratio test* AIC = 5,204, *p* < 0.001; Q5 AIC = 5,389, *p* < 0.001). The scores of the intraclass correlation coefficients are clearly related among themselves (Q4: ICC = 0.088; Q5: ICC = 0.369).

## Conclusion and discussion

Our study’s purpose was to compare the evolution of the learning process of three groups of 11–12-year-olds who were given a task to solve on the Collective Learning Platform, https://ic.kampal.com/ on the subject of popularity and social comparison. One of the groups was large (274 participants), and the other two were smaller (56 and 69 participants, respectively). The platform offers an innovative digital learning environment, as its design manages to combine individual work with work in small groups of five people, distributing the information and solutions produced by those small groups to all participants all across the network in each session, and ultimately generating a consensus solution based on the participants’ choices. We hypothesized that the number of participants in each learning session would affect how the activity evolved on the platform.

Results show that the production of answers and the participants’ user experience during the activity differed according to group size. The largest group evolved phase-by-phase in four of the five questions (in the three multiple-choice questions and one of the two open-ended questions requiring a written answer in text form). On Question 1 (multiple-choice), the Large Group improved its score (the mean of all participants) until its final score surpassed that of the other two groups, although it had started out with a score below theirs. The Large Group also presented a further salient characteristic. On Questions 4 and 5 (open questions requiring a textual written answer), Group 1’s total number of answers and the mean syntactical complexity of responses was greater than the two smaller groups, and those differences persisted throughout all seven phases of the activity. On the open-ended questions, we observe certain changes throughout the activity’s phases but find no evidence that those changes varied according to group. We thus conclude that the Large Group (Group 1) managed to optimize its performance on multiple-choice questions and on open-ended text under determined conditions; however, under other conditions, such differences do not appear, and Group 1’s pattern of change and evolution is similar to the pattern followed by Groups 2 and 3.

This result nevertheless requires detailed analysis since several relevant variables must be taken into consideration, some of which have already been introduced by authors such as Woolley et al. (2015), Woolley and Aggarwal (2020) when analyzing the factors that affect the development processes of collective intelligence, which form the conceptual basis of the Collective Learning platform. Those variables could help us grasp the underlying causes of some of the differences among the three groups. The first major difference among them is their degree of internal heterogeneity. The Large Group consists of students from 11 different schools, whereas the small groups are gathered from three classes of the same school. The Large Group’s greater degree of heterogeneity might explain why it has a lower-level point of departure on Question 1. It might also explain its greater degree of variability on the multiple-choice questions. However, this finding might be at odds with the greater degree of variance that one of the small groups presented on one of the open-ended questions. Moreover, the smaller groups wrote simple text in response to the open-ended questions, which might be a symptom of less motivation in tackling the task, or less competency in the domain they were dealing with.

The Large Group was thus a gathering from 11 different schools, whereas the small groups came from one school only; these differences in terms of heterogeneity and varying composition of groups might entail different sorts of dynamics during the activity. Such dynamics could be basically due to more assertive leadership styles in the small groups, where face-to-face interaction might have impinged upon online interaction on the platform. Leaders might have appeared, and their attitudes toward the assignment could have led to a reduction of activity in the remainder of the small group members. This possibility is manifest in the lower number of contributions made by the small groups on the open-ended questions: not only on Questions 4 and 5, but also in the explanatory text of Questions 1, 2, and 3 (see annex). Woolley et al. (2015) affirm that top-down factors are key in the emergence of collective intelligence: an ideal group is one in which participation is distributed equally among all participants, with only few well-defined leadership roles that might otherwise decrease the overall level of involvement. Jeffredo et al. (2024) also refer to these types of factors when analyzing emergent interaction conditions as being key to understanding how intelligence emerges in small groups. Derex et al. (2013) also cite this diversity of large groups as a condition for maintaining certain characteristics of cultural diversity. This supports the hypothesis that large groups can promote this heterogeneity and, consequently, cultural evolution. They experimentally test how group size directly affects performance. In any case, their groups operate in a different dimension to the one analyzed here, with a maximum of 18 participants.

It is equally difficult to explain the differences among the levels of points of departure in the three groups. The Large Group has a higher-level point of departure in all matters associated with implication in the task: i.e., the number of times participants record responses to the open-ended questions and the syntactical complexity of those responses. As the students in all three groups come from the same age group, our grounds for comparison might lie in differences in group composition from a socio-demographic perspective: the larger group would be more heterogeneous in this respect. The groups may also have approached the task with differing degrees of motivation. One of the conditions that was meant to motivate students was the chance to collaborate with students from other schools. Such differences in initial motivation might also be responsible for a greater degree of commitment to the task: with greater commitment, a learning opportunity is better exploited. Previous studies on collaboration have shown that preparation for collaboration is an important condition for its success (Eshuis et al., 2019; Mende et al., 2021). Moreover, successful groups maintain differentiated activity patterns (Barron, 2003; Liu et al., 2023; Zhang et al., 2023), as can be seen in the differences we observe among the groups in our study in terms of response syntactical complexity (production quantity).

To summarize at this point, the statistically significant differences we have observed might be due to differing degrees of heterogeneity within the groups and possible interactions that occurred outside the digital space.

Another condition that has the potential to affect results directly could be related to the type of task with which participants were confronted: specifically, response format. Part of the task we devised consisted of closed, multiple-choice answers. Such answers may be easier to quantify, but they have a direct effect on academic performance and on learning strategies. as previous studies in the field of education have shown (Attali et al., 2016; Fraundorf et al., 2023; Melovitz Vasan et al., 2018). The lack of evidence of changes in learning attitude in the small groups might thus have to do with the multiple-choice response option we chose for Questions 1, 2, and 3. In learning contexts, feedback provided by peers is more valuable in the form of open-ended responses than in closed (MC) responses (Attali et al., 2016). Indeed, in our sample, differences among groups are less pronounced on open-ended questions. Thus, all in all, the effect of response format and the degree of group homogeneity could be two key factors that help explain our results.

We should not ignore a further potential explanatory factor. An emerging hypothesis in the field of collective intelligence postulates that under determined conditions, larger groups of people might be able to resolve tasks more adequately. Although the relationship between size and performance is complex (Takefuji, 2023), it could explain the more notable progress achieved in our study by the Large Group on certain tasks. However, our finding cannot be compared with other studies that have compared results from large groups with those achieved by a small number of participants (de Back Tycho et al., 2021; Fay et al., 2000; Jeffredo et al., 2024). The latter authors found that groups of up to 5 people functioned well, but their performance decreased when the number of participants doubled (Fay et al., 2000). However, other projects have produced notable collective achievements with large groups, as can be seen in recent studies by Rosenberg et al. (2023, 2024) in the field of Artificial Swarm Intelligence. The latter authors have shown that a large group of 241 people can collaboratively solve complex tasks in an online environment that combines small and large groups to equal measure. However, they did not compare the results of groups of different sizes. Other work in simulation environments has attempted to optimize large-group work by managing interaction processes with experts (Bernstein et al., 2018; O’Bryan et al., 2025; Truong and Nguyen, 2024). However, the interaction model proposed by our platform differs in that it combines elements of small-group-oriented problem solving with a final large-group decision-making phase. The relevance of their method lies, as in ours, in the possibility of creating collaborative environments where participants can cooperate to build solutions to problems/tasks presented to them. This combination of processes can, in turn, reduce the negative impact of dispersion and the difficulty of obtaining consensus that is often found in large groups (Garzón et al., 2025; Kerr and Tindale, 2004).

At any rate, our study has certain limitations. These, in turn, can lead us to submit our conclusions to critical reflection. Participants were assigned on a non-random basis; thus, not all the variables in each group can be controlled. Differences among groups in terms of motivation can arise, and such differences are of a nature that would not be controllable under “natural” (face-to-face) conditions. Moreover, our results were obtained on the basis of only one learning task designed to help students develop socio-emotional competencies (Cebollero-Salinas et al., 2022a,2022b). We cannot guarantee that results might have been different if students had been given further types of tasks or activities to resolve. A third drawback is that our results depend on the type of measure we applied to evaluate participants’ responses. Thus, for instance, we opted for easily quantifiable multiple-choice answers on three of the five questions. We are nevertheless aware that such opting for multiple-choice can have an incidence on participants’ degree of activity; moreover, it can lead to problems associated with sensitivity to change. On the other hand, using response syntactical complexity as an indicator of participant activity can be a convenient option in our attempt to establish motivational patterns but does not guarantee that the content in those responses is necessarily of better quality. At any rate, closed response evaluation systems (multiple-choice) remain an economical option in terms of evaluation (Melovitz Vasan et al., 2018). In the near future, the use of generative artificial intelligence will most probably give rise to new situations that will allow researchers to automatize and much more rapidly evaluate large quantities of data obtained from digital interaction. Propositions in this sense have already been put forward by Ariely et al. (2023), Çinar et al. (2020), and Cress and Kimmerle (2023). By using ChatGPT 4.0, researchers could measure and evaluate large quantities of data while using the same AI tool to provide automatic feedback to learners; this, in turn, would have a direct incidence on the learning process.

In any case, our study also shows interesting strengths, particularly that of presenting a new learning environment with a vast potential for creating learning communities in digital environments, as certain authors have already pointed out (Klieger, 2016; Tenorio et al., 2021). This digital space makes it possible to break down the barriers of the classroom and combine the accumulated experience of large groups with the possibilities offered by artificial intelligence to create new interactive spaces that overcome traditional barriers of face-to-face interaction (Allam and Elyas, 2016). Designed on the basis of a groundbreaking theoretical framework (collective intelligence and complex systems), such spaces can serve as a valuable, innovative platform on which activities with added learning value can be carried out: for instance, as in our case, the improvement of socio-emotional competencies.

Finally, we wish to point out certain educational implications. Public policies could promote more valuable, in-depth, complex learning processes by basing them on collective intelligence. Interdisciplinary projects could generate collaborative networks that would not be limited by geographical location, thereby ensuring genuine heterogeneity and compensating for inequalities in the population by giving priority to collaboration with rural environments and with users from more or less vulnerable socioeconomic milieus. Moreover, online networks can expand collaboration among users to an international level.

Teachers, in their role as facilitators of quality learning experiences and agents of social change, can now resort to a methodologically useful tool they can apply in many different school subjects. In service-learning (S-E) environments implanted in heterogeneous communities, collective intelligence has the capacity to assist users in tackling complex problems and dealing with issues of increasing social relevance.

## Data Availability

The datasets presented in this study can be found in online repositories. This data can be found here: https://zenodo.org/records/15714969.

## Footnotes

1
http://linguistic-profiling.italianlp.it/

## References

[B1] Adjin-Tettey T. D. (2022). Teenagers and new media technologies: Gratifications obtained as a factor for adoption. *Commun. J. Commun. Stud. Afr.* 41:34. 10.36615/jcsa.v41i2.2239

[B2] Aggarwal I. Woolley A. W. Chabris C. F. Malone T. W. (2019). The impact of cognitive style diversity on implicit learning in teams. *Front. Psychol.* 10:112. 10.3389/fpsyg.2019.00112 30792672 PMC6374291

[B3] Allam M. Elyas T. (2016). Perceptions of using social media as an ELT tool among EFL teachers in the saudi context. *English Lang. Teach.* 9:1. 10.5539/elt.v9n7p1

[B4] Ariely M. Nazaretsky T. Alexandron G. (2023). Machine learning and hebrew NLP for automated assessment of open-ended questions in biology. *Int. J. Artif. Intell. Educ.* 33 1–34. 10.1007/s40593-021-00283-x

[B5] Attali Y. Laitusis C. Stone E. (2016). Differences in reaction to immediate feedback and opportunity to revise answers for multiple-choice and open-ended questions. *Educ. Psychol. Meas.* 76 787–802. 10.1177/0013164415612548 29795888 PMC5965532

[B6] Barron B. (2003). When smart groups fail. *J. Learn. Sci.* 12 307–359. 10.1207/S15327809JLS1203_1

[B7] Bautista P. Cano-Escoriaza J. Sánchez E. V. Cebollero-Salinas A. Orejudo S. (2022). Improving adolescent moral reasoning versus cyberbullying: An online big group experiment by means of collective intelligence. *Comp. Educ*. 189:104594. 10.1016/j.compedu.2022.104594

[B8] Becker J. Brackbill D. Centola D. (2017). Network dynamics of social influence in the wisdom of crowds. *Proc. Natl. Acad. Sci. U.S.A.* 114 E5070–E5076. 10.1073/pnas.1615978114 28607070 PMC5495222

[B9] Bernstein E. Shore J. Lazer D. (2018). How intermittent breaks in interaction improve collective intelligence. *Proc. Natl. Acad. Sci. U.S.A.* 35 8734–8739. 10.1073/pnas.1802407115 30104371 PMC6126746

[B10] Brunato D. Cimino A. Dell’Orletta F. Montemagni S. Venturi G. (2020). “Profiling-UD: A tool for linguistic profiling of texts,” in *Proceedings of the 12th edition of international conference on language resources and evaluation (LREC 2020)*, (Marseille), 11–16.

[B11] Cebollero-Salinas A. Bautista P. Cano J. Orejudo S. (2022c). E-competences and collective intelligence. Proposals for emotional development in online interactions. *Rev. Int. Educ. Emocional Bienestar* 2, 13–32. 10.48102/rieeb.2022.2.1.21

[B12] Cebollero-Salinas A. Cano-Escoriaza J. Orejudo S. (2022a). Social networks, emotions, and education: Design and validation of e-COM, a scale of socio-emotional interaction competencies among adolescents. *Sustainability* 14:2566. 10.3390/su14052566

[B13] Cebollero-Salinas A. Cano-Escoriaza J. Orejudo S. (2022b). Are emotional e-competencies a protective factor against habitual digital behaviors (media multitasking, cybergossip, phubbing) in Spanish students of secondary education? *Comp. Educ.* 181:4464. 10.1016/j.compedu.2022.104464

[B14] Cebollero-Salinas A. Elboj-Saso C. ñiguez-Berrozpe T. Bautista-Alcaine P. (2024a). Confronting fake news through collective intelligence in adolescents. *Int. J. Hum. Comp. Interact*. 41, 8122–8135. 10.1080/10447318.2024.2405567

[B15] Cebollero-Salinas A. Orejudo S. Cano-Escoriaza J. Íñiguez-Berrozpe T. (2022d). Cybergossip and problematic internet use in cyberaggression and cybervictimisation among adolescents. *Comp. Hum. Behav.* 131:107230. 10.1016/j.chb.2022.107230

[B16] Cebollero-Salinas A. Orejudo-Hernández S. Cano-Escoriaza J. (2024b). Cybergossip in adolescence: Its relationship with social competency, empathy, emotions in online communication and socio-emotional e-competencies by gender and age. *Cyberpsychol. J. Psychosoc. Res. Cyberspace* 18. 10.5817/CP2024-2-2

[B17] Chimbi G. T. Jita L. C. (2021). Resurgence of large class sizes and pedagogical reform in 21st century secondary school history classrooms. *Res. Soc. Sci. Technol.* 6 45–63. 10.46303/ressat.2021.24

[B18] Çinar A. Ince E. Gezer M. Yilmaz Ö (2020). Machine learning algorithm for grading open-ended physics questions in Turkish. *Educ. Inform. Technol.* 25 3821–3844. 10.1007/s10639-020-10128-0

[B19] Cress U. Kimmerle J. (2023). Co-constructing knowledge with generative AI tools: Reflections from a CSCL perspective. *Int. J. Comp. Supp. Collab. Learn.* 18 607–614. 10.1007/s11412-023-09409-w

[B20] de Back Tycho T. Tinga A. M. Louwerse M. M. (2021). CAVE-based immersive learning in undergraduate courses: Examining the effect of group size and time of application: Revista de universidad y sociedad del conocimiento. *Int. J. Educ. Technol. High. Educ.* 18:56. 10.1186/s41239-021-00288-5

[B21] Derex M. Beugin M. Godelle B. Raymond M. (2013). Experimental evidence for the influence of group size on cultural complexity. *Nature* 503 389–391. 10.1038/nature12774 24226775

[B22] Devine D. J. Philips J. L. (2001). Do smarter teams do better: A meta-analysis of cognitive ability and team performance. *Small Group Res.* 32 507–532. 10.1177/104649640103200501

[B23] Dhir A. Chen S. Nieminen M. (2017). Development and validation of the internet gratification scale for adolescents. *J. Psychoeduc. Assess.* 35 361–376. 10.1177/0734282916639460

[B24] Engel D. Woolley A. W. Jing L. X. Chabris C. F. Malone T. W. (2014). Reading the mind in the eyes or reading between the lines? Theory of mind predicts collective intelligence equally well online and face-to-face. *PLoS One* 9:e115212. 10.1371/journal.pone.0115212 25514387 PMC4267836

[B25] Erikson E. (1971). *Identidad, juventud y crisis.* Buenos Aires: Paidós.

[B26] Eshuis E. H. Judith T. V. Anjewierden A. Bollen L. Sikken J. Ton D. J. (2019). Improving the quality of vocational students’ collaboration and knowledge acquisition through instruction and joint reflection. *Int. J. Comp. Supp. Collab. Learn.* 14 53–76. 10.1007/s11412-019-09296-0

[B27] Fay N. Garrod S. Carletta J. (2000). Group discussion as interactive dialogue or as serial monologue: The influence of group size. *Psychol. Sci.* 11 481–486. 10.1111/1467-9280.00292 11202493

[B28] Festinger L. (1954). A theory of social comparison processes. *Hum. Relat.* 7 117–140. 10.1177/0018726754007002

[B29] Fraundorf S. H. Caddick Z. A. Nokes-Malach T. J. Rottman B. M. (2023). Cognitive perspectives on maintaining physicians’ medical expertise: III. Strengths and weaknesses of self-assessment. *Cogn. Res. Principles Impl.* 8:58. 10.1186/s41235-023-00511-z 37646932 PMC10469193

[B30] Fu R. Chen X. Liu J. Li D. (2018). Relations between social comparison orientation and adjustment in Chinese adolescents: Moderating effects of initial adjustment status. *Int. J. Psychol.* 53 133–141. 10.1002/ijop.12278 27168008

[B31] Gabarre-González J. Cano-Escoriaza J. Cebollero-Salinas A. (2024). Nomophobia and education: Formative challenges in gratifications in the use of the Internet and social comparison. A study according to sex. *Aloma* 42, 21–30. 10.51698/aloma.2024.42.2.21-30

[B32] Gallucci M. (2019). *GAMLj: General analyses for linear models. [jamovi module].* Available online at: https://gamlj.github.io/ (accessed April 05, 2024).

[B33] García-Jiménez A. López-Ayala-López M. C. Gaona-Pisionero C. (2012). A vision of uses and gratifications applied to the study of Internet use by adolescents. *Commun. Soc.* 25 231–254. 10.15581/003.25.36168

[B34] Garzón M. Álvarez-Pomar L. Rojas-Galeano S. (2025). From collective intelligence to global optimisation: An agent-based model approach. *Computing* 107:83. 10.1007/s00607-025-01429-8

[B35] Geifman D. Raban D. R. (2015). Collective problem-solving: The role of self-efficacy, skill, and prior knowledge. *Interdiscip. J. eSkills Lifelong Learn.* 11 159–178. 10.28945/2319

[B36] Geng J. Wang Y. Wang H. Wang P. Lei L. (2022). Social comparison orientation and cyberbullying perpetration and victimization: Roles of envy on social networking sites and body satisfaction. *J. Interpers. Violence* 37:3486. 10.1177/08862605211023486 34092135

[B37] Hair J. F. Anderson R. E. Tathum R. L. Black W. C. (2007). *Multivariate data analysis*, 5th Edn. New York, NY: McGraw Hill Publishing.

[B38] Hamada D. Nakayama M. Saiki J. (2020). Wisdom of crowds and collective decision-making in a survival situation with complex information integration. *Cogn. Res. Principles Impl.* 5:48. 10.1186/s41235-020-00248-z 33057843 PMC7561655

[B39] Jang K. Park N. Song H. (2016). Social comparison on Facebook: Its antecedents and psychological outcomes. *Comp. Hum. Behav.* 62 147–154. 10.1016/j.chb.2016.03.082

[B40] Jeffredo A. Clesse C. Batt M. (2024). Interpersonal factors that contribute to collective intelligence in small groups a qualitative systematic review. *Mind Soc.* 23 145–162. 10.1007/s11299-024-00307-8

[B41] Jeng Y. L. Huang Y. M. (2019). Dynamic learning paths framework based on collective intelligence from learners. *Comp. Hum. Behav.* 100 242–251. 10.1016/j.chb.2018.09.012

[B42] Kerr N. L. Tindale R. S. (2004). Group performance and decision making. *Annu. Rev. psychol.* 55 623–655. 10.1146/annurev.psych.55.090902.142009 14744229

[B43] Klieger A. (2016). The use of social networks to employ the wisdom of crowds for teaching. *TechTrends Link. Res. Pract. Improve Learn.* 60 124–128. 10.1007/s11528-016-0020-0

[B44] Kozhevnikov M. Evans C. Kosslyn S. M. (2014). Cognitive style as environmentally sensitive individual differences in cognition: A modern synthesis and applications in education, business, and management. *Psychol. Sci. Public Interest* 15 3–33. 10.1177/1529100614525555 26171827

[B45] Lewin K. M. Ellithorpe M. E. Meshi D. (2022). Social comparison and problematic social media use: Relationships between five different social media platforms and three different social comparison constructs. *Pers. Individ. Differ.* 199:1865. 10.1016/j.paid.2022.111865

[B46] Lins S. L. B. L. Campos M. Leite A. C. Carvalho C. L. Cardoso S. Natividade J. C. (2016). Evidências de validade da Escala de Orientação para a Comparação Social (INCOM) para o contexto de adolescentes portugueses. *Psicología* 30 1–14. 10.17575/rpsicol.v30i1.1034

[B47] Liu P. He J. Li A. (2019). Upward social comparison on social network sites and impulse buying: A moderated mediation model of negative affect and rumination. *Comp. Hum. Behav.* 96 133–140. 10.1016/j.chb.2019.02.003

[B48] Liu S. Kang L. Liu Z. Fang J. Yang Z. Sun J. (2023). Computer-supported collaborative concept mapping: The impact of students’ perceptions of collaboration on their knowledge understanding and behavioral patterns. *Interact. Learn. Environ.* 31 3340–3359. 10.1080/10494820.2021.1927115

[B49] Mao A. Winter M. Suri S. Watts D. J. (2016). An experimental study of team size and performance on a complex task. *PLoS One* 11:e0153048. 10.1371/journal.pone.0153048 27082239 PMC4833429

[B50] Melovitz Vasan C. A. DeFouw D. O. Holland B. K. Vasan N. S. (2018). Analysis of testing with multiple choice versus open-ended questions: Outcome-based observations in an anatomy course. *Anat. Sci. Educ.* 11 254–261. 10.1002/ase.1739 28941215

[B51] Mende S. Proske A. Narciss S. (2021). Individual preparation for collaborative learning: Systematic review and synthesis. *Educ. Psychol.* 56 29–53. 10.1080/00461520.2020.1828086

[B52] Miao H. Li Z. Yang Y. Guo C. (2018). Social comparison orientation and social adaptation among young Chinese adolescents: The mediating role of academic self-concept. *Front. Psychol.* 9:1067. 10.3389/fpsyg.2018.01067 29997555 PMC6030545

[B53] Navajas J. Niella T. Garbulsky G. Sigman M. (2018). Aggregated knowledge from a small number of debates outperforms the wisdom of large crowds. *Nat. Hum. Behav.* 2 126–132. 10.1038/s41562-017-0273-4

[B54] O’Bryan L. R. Oxendahl T. Garnier S. Segarra S. Wettergreen M. Sabharwal A. (2025). A novel approach to studying the role influence plays in team collective intelligence. *Collective Intell*. 4, 1–16. 10.1177/26339137251343584

[B55] Orejudo S. Cano-Escoriaza J. Cebollero-Salinas A. B. Bautista P. Clemente-Gallardo J. Rivero A. (2022). Evolutionary emergence of collective intelligence in large groups of students. *Front. Psychol*. 13:848048. 10.3389/fpsyg.2022.848048 36405219 PMC9666766

[B56] Orejudo S. Fernández Turrado T. Garrido Laparte M. Á (2008). Elaboración y trabajo con casos y otras metodologías activas: Cuatro experiencias de un grupo de profesores de la Facultad de Educación de Zaragoza. *Rev. Interunivers. Form. Profes.* 63 21–46.

[B57] Parks-Stamm E. J. Zafonte M. Palenque S. M. (2017). The effects of instructor participation and class size on student participation in an online class discussion forum. *Br. J. Educ. Technol.* 48 1250–1259. 10.1111/bjet.12512

[B58] Pescetelli N. Rutherford A. Rahwan I. (2021). Modularity and composite diversity affect the collective gathering of information online. *Nat. Commun.* 12:3195. 10.1038/s41467-021-23424-1 34045445 PMC8159948

[B59] Qiu M. Hewitt J. Brett C. (2012). Online class size, note reading, note writing and collaborative discourse. *Int. J. Comp. Supp. Collab. Learn.* 7 423–442. 10.1007/s11412-012-9151-2

[B60] Rosenberg L. Willcox G. Schumann H. (2023). Towards collective superintelligence, a pilot study. *arXiv* [Preprint]. arXiv:2311.00728. 10.48550/arXiv.2311.00728

[B61] Rosenberg L. Willcox G. Schumann H. Mani G. (2024). Towards collective superintelligence: Amplifying group IQ using conversational swarms. *arXiv* [Preprint]. arXiv:2401.15109. 10.48550/arXiv.2401.15109

[B62] Servidio R. Sinatra M. Griffiths M. D. Monacis L. (2021). Social comparison orientation and fear of missing out as mediators between self-concept clarity and problematic smartphone use. *Addict. Behav.* 122:107014. 10.1016/j.addbeh.2021.107014 34153569

[B63] Shaw R. (2013). The relationships among group size, participation, and performance of programming language learning supported with online forums. *Comp. Educ.* 62 196–207. 10.1016/j.compedu.2012.11.001

[B64] Siqin T. van Aalst J. Chu S. K. Wah. (2015). Fixed group and opportunistic collaboration in a CSCL environment. *Int. J. Comp. Supp. Collab. Learn.* 10 161–181. 10.1007/s11412-014-9206-7

[B65] Sorensen C. (2015). An examination of the relationship between online class size and instructor performance. *J. Educ. Online* 12 140–159. 10.9743/JEO.2015.1.3

[B66] Suls J. Martin R. Wheeler L. (2002). Social comparison: Why, with whom, and with what effect? *Curr. Dir. Psychol. Sci.* 11 159–163. 10.1111/1467-8721.00191

[B67] Takefuji Y. (2023). Why the power of diversity does not always produce better groups and societies. *Bio Syst.* 229:104918. 10.1016/j.biosystems.2023.104918 37196894

[B68] Tenorio T. Isotani S. Bittencourt I. I. Lu Y. (2021). The state-of-the-art on collective intelligence in online educational technologies. *IEEE Trans. Learn. Technol.* 14 257–271. 10.1109/TLT.2021.3073559

[B69] The jamovi project, (2023). *Jamovi*. *(Version 2.4) [Computer Software].* Available online at: https://www.jamovi.org (accessed January 15, 2024).

[B70] Topolewska-Siedzik E. Cieciuch J. (2018). Trajectories of identity formation modes and their personality context in adolescence. *J. Youth Adolesc.* 47 775–792. 10.1007/s10964-018-0824-7 29492870 PMC5852173

[B71] Toyokawa W. Gaissmaier W. (2022). Conformist social learning leads to self-organised prevention against adverse bias in risky decision making. *eLife* 11:e75308. 10.7554/eLife.75308 35535494 PMC9090329

[B72] Toyokawa W. Whalen A. Laland K. N. (2019). Social learning strategies regulate the wisdom and madness of interactive crowds. *Nat. Hum. Behav.* 3 183–193. 10.1038/s41562-018-0518-x 30944445

[B73] Truong H. B. Nguyen V. D. (2024). A new method for enhancing collective intelligence using expert’s knowledge. *J. Inform. Telecommun.* 8 531–547. 10.1080/24751839.2024.2318073

[B74] Vogel E. A. Rose J. P. Okdie B. M. Eckles K. Franz B. (2015). Who compares and despairs? The effect of social comparison orientation on social media use and its outcomes. *Pers. Individ. Differ.* 86 249–256. 10.1016/j.paid.2015.06.026

[B75] Woolley A. W. Aggarwal I. Malone T. W. (2015). Collective intelligence and group performance. *Curr. Dir. Psychol. Sci.* 24 420–424. 10.1177/0963721415599543

[B76] Woolley A. Aggarwal I. (2020). “Collective intelligence and group learning,” in *The Oxford handbook of group and organizational learning, Oxford library of psychology*, eds Argote L. Levine J. M. (Oxford: Oxford Academic), 10.1093/oxfordhb/9780190263362.013.46

[B77] Woolley A. Chabris C. Pentland A. Hashmi N. Malone T. W. (2010). Evidence for a collective intelligence factor in the performance of human groups. *Science* 330 686–688. 10.1126/science.1193147 20929725

[B78] Yahosseini K. S. Moussaïd M. (2020). Comparing groups of independent solvers and transmission chains as methods for collective problem-solving. *Sci. Rep.* 10:3060. 10.1038/s41598-020-59946-9 32080278 PMC7033214

[B79] Zhang L. Pan M. Yu S. Chen L. Zhang J. (2023). Evaluation of a student-centered online one-to-one tutoring system. *Interact. Learn. Environ.* 31 4251–4269. 10.1080/10494820.2021.1958234

